# Gibberellin deficiency is responsible for shy-flowering nature of *Epipremnum aureum*

**DOI:** 10.1038/srep28598

**Published:** 2016-06-27

**Authors:** Chiu-Yueh Hung, Jie Qiu, Ying-Hsuan Sun, Jianjun Chen, Farooqahmed S. Kittur, Richard J. Henny, Gulei Jin, Longjiang Fan, Jiahua Xie

**Affiliations:** 1 Department of Pharmaceutical Sciences, Biomanufacturing Research Institute & Technology Enterprise, North Carolina Central University, Durham, NC 27707, USA; 2 Department of Agronomy, Zhejiang University, Hangzhou 310029, China; 3 Department of Forestry, National Chung Hsing University, Taichung 402, Taiwan; 4 Environmental Horticulture Department and Mid-Florida Research and Education Center, Apopka, University of Florida, Apopka, FL 32703, USA

## Abstract

*Epipremnum aureum* is an extremely popular houseplant belonging to the Araceae family of angiosperms, but it does not flower either in the wild or under cultivation. We uncovered the potential causes of its shy-flowering nature by building the transcriptome using next-generation sequencing and identifying floral-related genes that are differentially expressed between vertical growth (VG, adult) and horizontal growth (HG, juvenile) plants. Transcripts of the gibberellin (GA) biosynthetic gene *EaGA3ox1* and GA-responsive floral meristem identity gene *EaLFY* were absent in both VG and HG plants, suggesting that a deficiency of bioactive GAs may be responsible for its shy-flowering nature. This hypothesis is supported by undetectable or low levels of three bioactive GAs, and exogenous GA_3_ triggered flowering in both plants. Our study resolves the mystery why *E. aureum* fails to flower, and reveals the positive role of GAs in floral transition in perennials.

Flowering is a remarkable characteristic of the angiosperms (flowering plants) for reproductive success^1^,^2^,^3^. In nature, angiosperms flower at least once in their lifetime. Even seldom-flowering monocarpic perennials, such as bamboo and American agave, grow vegetatively for many years then bloom once near the end of their life cycle. However, *Epipremnum aureum* described as “shy-flowering” plants^4^ fails to flower irrespective of where (wild or cultivated) and how (vertically versus horizontally) it grows^4^,^5^. *E. aureum* propagated vegetatively is one of the most popularly grown houseplants worldwide with different nick names in different places, such as ‘Hunter’s Robe’, ‘Devil’s Ivy’, ‘Ivy Arum’ and ‘Silver Vine’ in North American, ‘Golden Vine’ in China and ‘Money Plant’ in India^5^,^6^. Although it is so popular with several different color defective varieties^4^,^5^,^6^, its shy-flowering nature remains a mystery, and this mystery is hard to solve by traditional hybridization or direct gene comparison between a shy-flowering mutant and a wild type. Thus far, molecular defects responsible for its shy-flowering nature remain elusive.

*E. aureum* is a perennial vine species native to French Polynesia and a member of the Araceae^6^,^7^. Araceae is a large and diverse family with 3,800 published species in 120 genera^8^, but no other shy-flowering species except *E. aureum* has been reported yet^4^. Flowers of this family are borne on a type of inflorescence called a spadix, which is an important feature used for the classification of any given species within the family^8^. Because of shy-flowering nature, *E. aureum* had a winding history of its nomenclature^4^. *E. aureum* was first classified as *Pothos aureus* based on juvenile materials in 1880^9^. Even today, ‘Pothos’ is still a common name used for this species. Based on the overall appearance of mature plants, *E. aureum* was renamed as *Scindapsus aureus*^10^. When its flowers were first observed in 1962, this widely cultivated species was given a new name as *Raphidophora aurea*^11^. Owing to its flower highly similar to that of *E. pinnatum,* it was re-classified into *E. pinnatum* in 1963^7^. Only after more careful observation of its flower and other characters including leaf shape and lamina, sheath and growing speed, it was separated from *E. pinnatum* and named as *E. aureum* in 1964^4^. This long and tortuous history of its nomenclature also indicates that this species rarely flowers. Since 1962 there is no report of *E. aureum* flowering both in wild as well as in cultivation.

This shy-flowering nature in *E. aureum* suggests that its floral transition from vegetative to reproductive growth may be defective. Genes controlling floral transition have been studied intensively in annuals. In *Arabidopsis*, floral transition is known to be regulated by a complex integrated gene network involving photoperiod, vernalization, autonomous, age-related and GA pathways although the full picture of their coordination has not been revealed^12^,^13^,^14^,^15^,^16^. The activities of these pathway genes converge on a small number of floral integrators, such as *SOC1* and *AGL24*, which then activate the master flower identity regulator genes *LFY* and *AP1*^12^,^13^,^15^,^16^. Studies in *Arabidopsis* and other species have also found that most of the flower-related genes are conserved across species^13^,^16^. Although the current understanding of floral transition in biennials and perennials is limited^16^,^17^, the wealth of information about floral transition in *Arabidopsis* could be exploited to investigate the shy-flowering nature in *E. aureum*.

During floral transition studies, it was observed that entering its adult stage is a prerequisite for a plant to respond to floral inductive signals^12^,^18^. Horizontal growth (HG) *E. aureum* plants bearing small leaves are considered as juveniles whereas vertical growth (VG) plants with leaves several times larger are considered as adults^4^. *E. aureum’s* closest relative *E. pinnatum* flowers profusely under VG conditions^4^. Therefore, *E. aureum* HG and VG plants could be a valuable pair of materials to be used to unravel the mystery concerning its shy-flowering nature.

Like the most non-model plants, very little molecular work has been done in *E. aureum.* Only few genes have been cloned and characterized to determine its evolutionary position^19^ and to understand its variegated leaf formation^20^,^21^. To uncover its shy-flowering nature, we first applied next generation sequencing technology, which enables to generate transcriptomic sequences without known genome information^22^, to build its *de novo* transcriptome. Then we referenced to Arabidopsis floral-related gene information to investigate the molecular basis of shy-flowering using HG and VG plants. We discovered that the shy-flowering nature of *E. aureum* is caused by deficiency of phytohormone gibberellic acids (GAs).

## Results

### *E. aureum* has mixed characters of monocots and dicots

**Figure 1:**
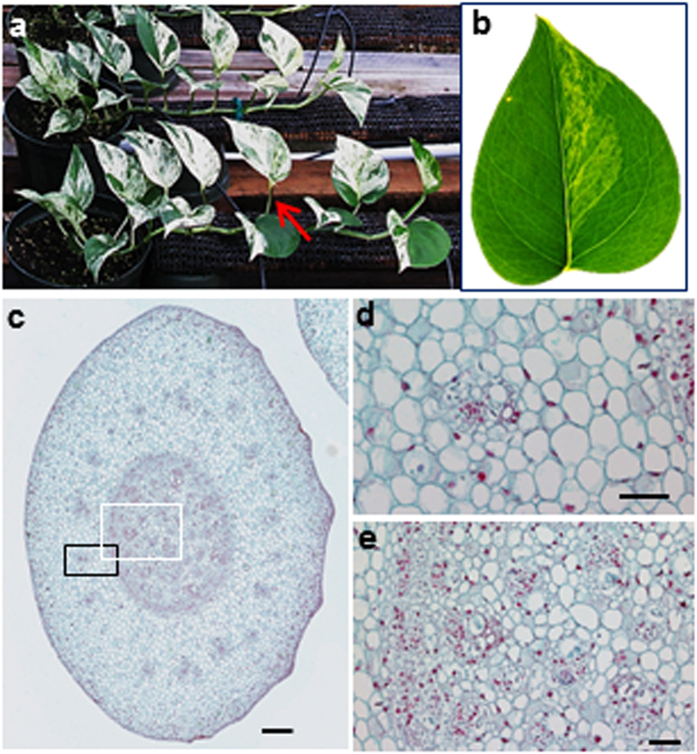
Morphology of *E. aureum*. (**a**) A variegated variety of ‘Marble Queen’ plant showing green and white leaf sectors. Red arrow indicates a petiole. (**b**) Its fully expanded leaf shows a typical venation pattern commonly observed in dicots. (**c**) A cross section of its stem shows ‘compound’ vascular bundles distributed in two separate layers: a ring in the outer layer and scattered in the inner layer. Scale bar = 200 μm. (**d**) Detailed vascular bundles for outer layer (black box in c.). Scale bar = 50 μm. (**e**) Detailed vascular bundles for inner layer (white box in c.). Scale bar = 50 μm.

Araceae was classified as an early-diverging monocot but with many characters not fitting in a typical monocot^23^,^24^. To understand *E. aureum* in detail, some characters were observed. *E. aureum* plant has mixed characters with a wide-blade leaf, an obvious petiole, a clear primary midvein and lateral secondary veins connected by arches close to the leaf margin area (Fig. 1a,b), which are distinct from most monocots having a narrow leaf blade and parallel main veins without an obvious petiole^1^,^23^. Its young stem has ‘compound’ vascular bundles^25^ organized in two separate layers (Fig. 1c). Those in the outer layer are arranged in a ring (Fig. 1c,d) similar to the pattern observed in dicots^26^, whereas those in the inner layer are scattered (Fig. 1c,e) following the pattern in most monocots^1^,^23^. These observations indicate that *E. aureum* likes the other Araceae members having some mixed dicot characters.

### Transcriptome of *E. aureum*

To identify the molecular basis of its shy-flowering nature, we built its transcriptome using the variegated variety called ‘Marble Queen’ (Fig. 1a) in order to have broad coverage because most of *E. aureum* varieties are variegated^4^,^5^. Both 454 and Illumina sequencing data (Supplementary Tables S1a,b) were employed for *de novo* transcriptome assembly because the hybrid 454/Illumina assemblies had better transcriptome and individual gene coverage^27^. For accuracy of assembly, its ploidy was determined by counting chromosomes. The results showed 60 chromosomes (Supplementary Fig. S1), which is the same number found in other diploid members of the genus *Epipremnum*^28^. A total of 41,059 contigs were assembled with an average length of 1,049 bp (Supplementary Table S1c) while 23,399 (57%) (Bioproject accession number: PRJNA286034) had homologs in public databases of plants.

### *E. aureum* is an early monocot

**Figure 2:**
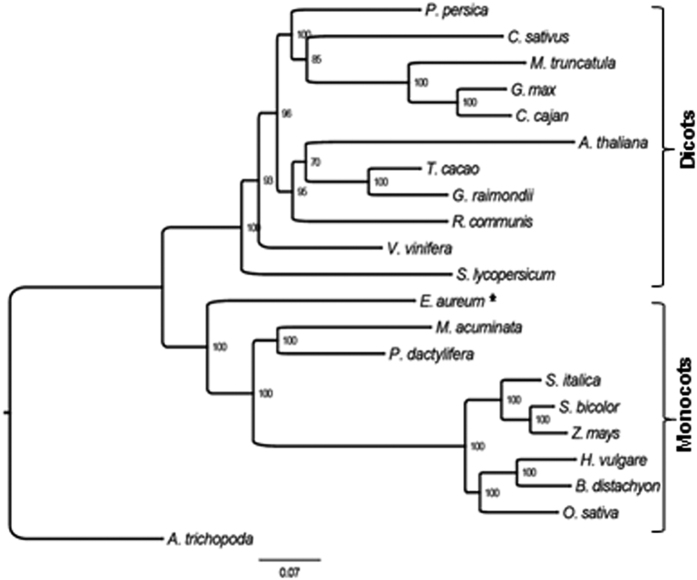
Evolutionary position of *E. aureum*. Phylogenetic tree of *E. aureum* (star) and other 20 genome sequenced plant species based on their 263 single copy orthologs.

In order to further determine its evolutionary position, *E. aureum* transcriptome together with 20 publicly available angiosperm genomes (Supplementary Table S2) were used to identify 263 orthologs (Supplementary Table S3) to build a phylogenetic tree with *Amborella trichopoda* as an out-group^1^,^3^. Phylogenetic analysis showed that *E. aureum* appeared in an isolated group of monocot species, but was close to dicots (Fig. 2). Of the remaining eight monocots, *Phoenix dactylifera* and *Musa acuminata* from Commelinids appeared as one group while six species from Poaceae formed another group but separated as C3 and C4 species. These results indicate that *E. aureum* is an early monocot and should be a flowering species.

### Differential expression of flower-related genes in VG and HG plants

**Figure 3:**
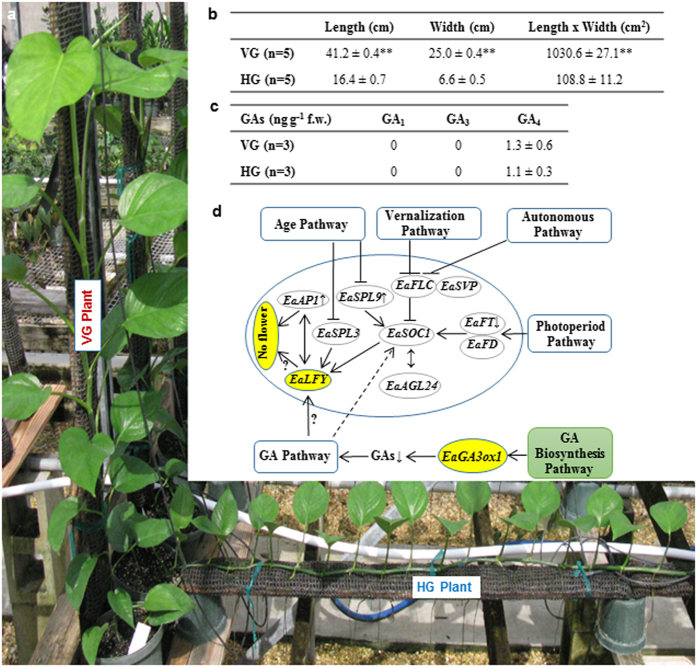
Studies of VG and HG ‘Jade’ plants. (**a**) VG and HG plants were maintained side-by-side in a greenhouse. (**b**) Difference in leaf sizes between VG and HG plants. **P < 0.01. (**c**) Contents of GAs in VG and HG shoot apexes. f.w.: fresh weight. (**d**) Expression patterns of some key genes from flowering inductive and GA biosynthesis pathways in VG compared to HG plants. Undetected genes *EaLFY* and *EaGA3ox1* are highlighted in yellow. Relationship of these key genes indicated with solid lines was adopted from Albani and Coupland (2010). Arrows indicate promoting events whereas T symbols denote repressing events on flowering. The dotted line means an inconclusive relationship. Unmarked genes represent no change; small ↑: increased; small **↓**: reduced; ?: potential involvement; GAs: GA_1_, GA_3_ and GA_4_.

Entering an adult stage is considered as a prerequisite for floral transition^12^,^18^. *E. aureum* VG plants bearing leaves several times larger than those in HG plants are considered to be adults^4^. Since most of the flower-related genes are conserved across species^13^,^16^, we therefore compared the expressions of 147 orthologs of flower-related genes from five floral inductive pathways of *Arabidopsis* (Supplementary Table S4) between HG and VG shoot transcripts (Supplementary Table S5), which were created using Illumina sequencing techniques. To avoid interference from variegated tissues on gene expression analysis, the green plant ‘Jade’ (Fig. 3a), a reversion of ‘Marble Queen’^4^, was used.

In the age pathway, the expression of *SPLs* is known to increase in *Arabidopsis*^29^ while the *TOE1* decreases^30^ after entering adult stage. Several *EaSPLs*, and subsequently several floral identity genes, had higher expressions accompanying lower expression of *EaTOE1* in VG compared with HG plants (Supplementary Table S4), suggesting that VG plants were indeed adult and better prepared for flowering. This is also supported by lower expressions of *EaGNC* and *EaGNL*, two flowering repressor orthologs *GNC* and *GNL* from the GA pathway^31^. Despite having adult characteristics, VG plants still did not flower, implying that one or more other factor(s) are required for flowering in *E. aureum*.

Examination of autonomous and vernalization pathway genes revealed that all genes had similar expression levels in VG and HG plants (Supplementary Table S4). Most of the key genes in the photoperiod pathway also appeared to have no differential expression. In the photoperiod pathway, FT interacts with FD to activate *SOC1* and they should have higher expression levels in adult plants than juveniles^12^,^32^. Conversely, transcripts of *EaFT*/*EaTSF* along with *EaFKF1* and *EaGI* were significantly lower in VG than HG plants. In addition, the expression of *EaSOC1* was similar in VG and HG plants. Since *SOC1* is convergently controlled by floral induction signals from the photoperiod, vernalization and autonomous pathways^15^,^16^, similar expression levels of *EaSOC1* in both plants together with the gene expression patterns related to these three pathways imply that none of the pathways were defective or responsible for lack of flowering.

The roles of GAs on floral initiation are complex. They promote flowering in annual and biennial species but inhibit flowering in perennials^14^. The promotion of flowering by GAs in *Arabidopsis* is thought to directly induce *LFY* and *SOC1* expressions^14^,^33^,^34^,^35^ as well as activate the *SPLs* via degradation of DELLAs^36^. Our results showed that expression of all DELLA ortholog members was equally high between VG and HG plants (Supplementary Table S4). Moreover, the *EaLFY* transcript could not be found and another GA-responsive gene *EaFPF1* was undetectable in either type of shoot. These results led us to hypothesize that bioactive GAs might be low, which led us to compare GA biosynthesis genes.

### Differential expression of GA biosynthesis genes and three bioactive GAs in VG and HG plants

To determine any impaired GA biosynthesis gene in *E. aureum*, the expressions of 24 orthologs of *Arabidopsis* GA biosynthesis genes were compared (Supplementary Table S6). *EaGA3ox1* encoding an enzyme involved in biosynthesis of bioactive GAs was not detected in either VG or HG plants. Among 24 orthologs, there were no major genes whose expression differed between VG and HG plants including undetected gene *EaGA3ox1*. To investigate the consequences of a lack of *EaGA3ox1* transcripts in both plants, we measured the levels of all three bioactive GAs in shoot apexes. GA_1_ and GA_3_ were not detected at all while only low levels of GA_4_ were detected (Fig. 3c), and were ~100-fold lower than that in *Arabidopsis*^37^.

The above differentially expressed gene (DEG) results were validated by qRT-PCR analysis with five selected genes from each pathway or group. Expression patterns of those validated genes matched 100% (Supplementary Fig. S2 and Tables S4 and S6). In summary, transcriptomic and GA analyses led us to believe that the deficiency of bioactive GAs could be responsible for shy-flowering phenomenon (Fig. 3d), and that application of GA should induce flowering at least in VG plants.

### Induction of flowering in both VG and HG plants by GA_3_ treatment

**Figure 4:**
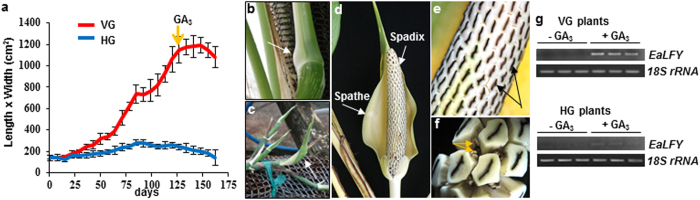
GA_3_ induced flowering and *EaLFY* expression. (**a**) The size (length x width (cm^2^)) of the young fully expanded leaf was recorded weekly. Data plotted were the average (n = 5) ±s.d. Arrow indicates the time of GA_3_ treatment. (**b**) Appearance of flower bud (white arrow) in VG plants. (**c**) Appearance of small flower bud (white arrow) in HG plants. (**d**) Inflorescence consisting of a leaf-shaped spathe and a spadix. (**e**) Close-up of inflorescence with many small, pentacyclic and prism-shaped female flowers (black arrows) tightly packed together. (**f**) Stamen of a male flower with anthers (yellow arrows) hidden between female flowers. (**g**) RT-PCR results of *EaLFY* and *18s rRNA* from three GA_3_ treated (+GA_3_) and three untreated (−GA_3_) VG (upper panel) and HG (lower panel) plants.

To test the above hypothesis, we sprayed 2,500 mg l^−1^ of GA_3_ on both VG and HG plants grown under the same conditions when VG plants had 4.5-fold larger leaves than HG ones (Fig. 4a and Supplementary Fig. S3b). Surprisingly, flower buds appeared first in HG plants 7 weeks after treatment (Fig. 4c) and then in VG after 8 weeks (Fig. 4b). Each plant produced one to three typical Araceae inflorescences (Fig. 4d). Inflorescences from HG were much smaller than those from VG plants. Each inflorescence was a spadix with many small, prism-shaped flowers tightly packed together (Fig. 4e,f). These results show that low levels of GAs were responsible for shy-flowering in *E. aureum*.

### Effects of exogenous GA_3_ treatment on each pathway

**Figure 5:**
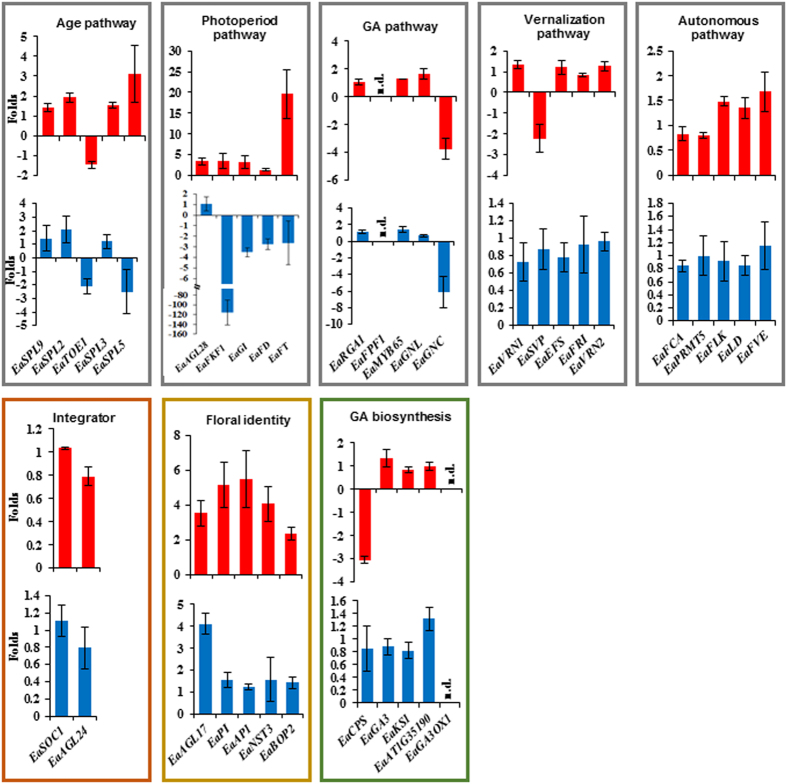
Results of qRT-PCR on selected genes from each pathway or group after treating with GA_3_ in VG (red) and HG (blue) plants. Data plotted are the fold changes representing the relative difference in expression between GA_3_ treated and untreated (as 1) samples. A positive value indicates induction while a negative value indicates reduction after GA_3_ treatment. Data shown are the average (n = 3 pairs of VG and HG plants) ±s.d. n.d.: not detected.

To better understand how exogenous GA_3_ promoted flowering, expression levels of five selected genes from each regulatory pathway or group were further analyzed. Only *EaGNC* in the GA pathway was found to be suppressed and floral meristem identity gene *EaAGL17* was induced significantly in both HG and VG plants (Fig. 5). The remaining four floral meristem identity genes were all induced, but only significantly in VG plants. In *Arabidopsis*, *LFY* is a plant-specific transcription factor to trigger the floral transition^38^,^39^ and the coordinated induction of *LFY* and *AP1* is decisive for floral initiation^34^,^40^. Because no *EaLFY* was detected in the original transcriptome, its partial genomic DNA sequences were cloned (Supplementary Fig. S4) to design primers for detecting its transcripts using RT-PCR. We observed that *EaLFY* was induced by GA_3_ treatment in both VG and HG plants (Fig. 4g), indicating that low levels of bioactive GAs were responsible for the absence of *EaLFY* transcript.

Additionally, four out of five genes from the photoperiod pathway and one from the age pathway were induced in VG but suppressed in HG plants following GA_3_ treatment (Fig. 5). These results together with unequal induced levels of floral meristem identity genes in VG and HG plants infer that these plants respond to GA_3_ differently. Of the other pathway genes examined, all five genes in the autonomous pathway did not respond significantly to GA_3_, and only *EaVSP* in the vernalization pathway was reduced ~2-fold in VG plants. In the GA biosynthetic pathway, only expression of *EaCPS* was inhibited significantly in VG plants. *EaGA3ox1* and the GA-responsive gene *EaFPF1* were still undetectable (Fig. 5).

## Discussion

Despite the fact that *E. aureum* is so popular and grows everywhere, its flowers are rarely observed with only one report in 1962^4^,^11^. As a result of its shy-flowering nature, no hybridization is possible to conduct traditional genetic studies to understand why this species rarely flowers. Utilizing the next generation sequencing techniques and employing floral-related gene information from model plant *Arabidopsis*, we have successfully uncovered that failure to flower in *E. aureum* is due to a lack of bioactive GAs, as the result of impairment of *EaGA3ox1*. This conclusion is supported by following evidences: 1) no detectable expression of the GA biosynthetic gene *EaGA3ox1* (Supplementary Table S6), 2) undetectable or low levels of bioactive GAs (Fig. 3c), 3) no expression of GA-responsive floral identity gene *EaLFY* (Supplementary Table S4), and 4) successful induction of flowering with exogenous GA (Fig. 4). In nature, there are still many plant species lacking genomic and genetic information, but they are economically or scientifically important. Our study provides a good example of how to employ available information from model species together with powerful next generation sequencing techniques to unravel previously unsolved mysteries of nature.

Studies on vegetative to reproductive switch in *Arabidopsis* reveal that the floral transition is regulated complexly by a network involving photoperiod, vernalization, autonomous, age-related and GA pathways^12^,^14^,^15^,^16^. Among these inductive pathways, the roles of GAs on floral initiation are the most complex. It has been reported that GAs promote flowering in annual and biennial species but inhibit flowering in perennials^14^. In perennials, inhibitory effects of GAs on flowering were established by the reduction of inflorescence numbers in grapevine when it was treated with exogenous GAs^41^. Nevertheless, our results show that low levels of GAs were responsible for shy-flowering in *E. aureum*, and that treatment with GAs could bypass other regulatory pathways to promote floral transition as previously suggested^33^,^34^. Our results contradict the previous report that GAs inhibit flowering in the perennial grapevine^41^. Recently, the roles of GAs in *Arabidopsis* were further divided into two phases – phase I promoting floral transition, followed inhibition of flower formation in phase II^42^. Our study together with the report by Yamaguchi *et al*.^42^ indicates that GAs can promote floral transition in *E. aureum* and possibly other perennials.

It was also noticed that VG and HG plants responded to GA_3_ treatment differently. Four (*EaFKF1*, *EaGI*, *EaFD* and *EaFT*) out of five genes from the photoperiod pathway and *EaSPL5* from the age pathway analyzed had same response patterns to GA_3_ treatment. They were induced in VG but suppressed in HG plants (Fig. 5). Their expressions together with floral meristem identity gene expression results (Fig. 5) imply that plants under different growing conditions react to GA_3_ treatment differently. It is understandable that these photoperiod pathway genes were induced in VG plants after GA_3_ treatment because their *Arabidopsis* orthologs are also known to be dramatically induced to promote flowering^12^,^32^. However, why GA_3_ treatment lowered the expression levels of these genes in HG plants is not clear yet? One major difference between VG and the HG plants is that the former has several fold larger leaves than the latter (Supplementary Fig. S3b). Because both of them could be induced by GA_3_ treatment to flower (Fig. 4), one possibility is that the expression levels of photoperiod genes selected may play roles in floral organ development since inflorescences from HG were much smaller than those from VG plants. Induction of GA pathway may bypass some of the other regulatory pathways to induce flowering. Understanding how GA bypasses other regulatory pathways of floral transition and organ development is important and warrants future investigation, but is beyond the scope of the current study.

In summary, we have demonstrated that failure to flower in *E. aureum* is due to a lack of bioactive GAs, as the result of impairment of *EaGA3ox1*. GA_3_ treatment can bypass other floral regulatory pathways to induce flowers. In *Arabidopsis*, GA is known to promote floral transition by repressing *GNC* to activate *SOC1*, which in turn activates *LFY* and *AP1*^31^. The mode of GA action on floral transition in *E. aureum* resembles that in *Arabidopsis*^31^ by inhibiting the activity of repressor *EaGNC*, resulting in induction of *EaAGL17*, followed by activation of *EaLFY*, *EaAP1* and other floral meristem identity genes. *AGL17* was known to promote floral transition via up-regulation of *LFY* and *AP1*^43^.

## Methods

### Plant materials, growth conditions and GA_3_ treatment

Variegated *E. aureum* ‘Marble Queen’ plants grown in soil under 23 °C and ~100 μmol m^−2^ s^−1^ light intensity were used for obtaining transcriptome sequences. In order to build a widely covered transcriptome, both green and white leaf tissues as well as other tissues were included. Specifically, RNA was prepared from young shoots including leaves, petioles and stems as well as young roots for 454 sequencing. For Illumina sequencing, equal amounts of green (MG) and white (MW) sectors from the same first expended young leaf were harvested for RNA isolation. All harvested tissues were frozen at −80 °C before RNA isolation. The same type of plant was also used for following studies. Young stems close to the first node were used for their histological study while young root tips were used for determining chromosome number. To study venation pattern, first fully expended young leaf with mostly green area was chosen for obtaining a better contrast image of veins.

For vertical and horizontal growth comparison and GA treatment studies, *E. aureum* ‘Jade’ plants with complete green leaves were used instead of variegated plants to avoid the possibility of differential gene expressions in different colored tissues. ‘Jade’ is a reversion of ‘Marble Queen’^4^. Young plants at the 5 to 6 leaf stage were used to set up for vertical and horizontal growth on Totem Poles as showed in Supplementary Fig. S3a. They were grown in a shaded greenhouse under a light intensity of ~450 μmol m^−2^ s^−1^ at the University of Florida’s Mid-Florida Research and Education Center, Apopka, FL, USA. When VG plants produced leaf sizes approximately 9.5-fold larger than those on HG plants (Fig. 3b), shoot apexes, as circled in Supplementary Fig. S5a, from both VG and HG plants were harvested to obtain young shoots for RNA isolation, and subsequent Illumina and qRT-PCR analyses. The same harvested tissues were also used for GA measurement. Plants with the same growth setting were used for GA treatment when VG plants reached leaf sizes approximately 4.5-fold larger than those in HG plants (Fig. 3a; Supplementary Fig. S3b). GA_3_ was chosen because it is as active as GA_4_, but more stable^37^. GA_3_ (GibGro 4% GA_3_ liquid, Agtrol Chemical Products) was dissolved in water to a final concentration of 2,500 mg l^−1^ and 0.02% Tween-20^5^. Each plant was sprayed once till shoot and all leaves were completely wet. After spraying, they were maintained under regular growth conditions described above. Shoot apexes before flower bud emerged from each plant, as circled in Supplementary Fig. S5b, were harvested to obtain young shoots in liquid nitrogen for RNA isolation, and subsequent qRT-PCR analysis.

### Genome data sources

Publicly available genome sequences of 20 plant species (Supplementary Table S2) were retrieved for present study. They included 11 dicots (*Arabidopsis thaliana*, *Cajanus cajan*, *Cucumis sativus*, *Glycine max*, *Gossypium raimondii*, *Mediucago truncatula*, *Prunus persica*, *Ricinus communis*, *Solanum lycopersicum*, *Theobroma cacao* and *Vitis vinifera*), eight monocots (*Brachypodium distachyon*, *Hordeum vulgare*, *Musa acuminate*, *Oryza sativa*, *Phoenix dactylifera*, *Sorghum bicolor*, *Setaria italica* and *Zea mays*) and one single sister species *Amborella trichopoda* to all other extant angiosperms^1^,^3^ used as an out-group. The sequences of four species were downloaded from independent websites - *P. dactylifera* (http://www.kacst.edu.sa/en/depts/jcg/ researchwork/Pages/default.aspx#dpgp), *A. trichopoda* (http://amborella.huck.psu.edu/ downloads/), *M. acuminate* (http://banana-genome.cirad.fr/download) and *H. vulgare* (ftp://ftpmips.helmholtz-muenchen.de/plants/barley/public_ data/). The sequences of the remaining plant species used in this study were downloaded from Phytozome (ftp://ftp.jgi-psf.org/pub/compgen/phytozome/v9.0/).

### Related *Arabidopsis* genes used

A total of 147 *Arabidopsis* flower-related genes and 24 GA biosynthesis pathway genes were used for the current study. Their full names and functional descriptions, and matched *E. aureum* contigs are listed in Supplementary Table S7. The selection of flower-related genes and the classification of their functional pathway/group were mainly based on Kim *et al*.^44^ with the consideration of other publications^12^,^13^,^14^,^15^,^16^,^17^,^32^,^45^ since many genes have more than one function involved in different regulatory pathways. All GA biosynthesis pathway genes were from http://pmn.plantcyc.org/ARA/NEW-IMAGE?object=GIBBERELLINS-BIOSYNTHESIS.

### Morphological observation and histological analysis

The fully expanded healthy leaves were used for observing leaf venation patterns, and their images were captured using a Nikon DX camera (Nikon Inc.). For histological analysis, young stems were first fixed in FAA solution (50% ethanol, 5% acetic acid and 4% formaldehyde) for 16 h and then dehydrated under a series of increasing ethanol concentrations. After xylene rinsing, they were embedded in paraffin wax. A Leica RM2145-microtome (Leica Microsystems) was used for preparing 5 μm sections. After immobilizing on slides, the deparaffinized specimens were stained with Johansen’s Safranin and Fast Green stain^46^. The stained images were captured with Zeiss-Axio Imager M2 (Zeiss). The images were analyzed and stitched using Zeiss Zen 2012 (Zeiss).

### Determination of chromosome number

In order to observe the somatic chromosomes, excised root tips were first prepared and treated using the protocol as described in Hung *et al*.^47^. The chromosome images were observed under a light microscope (Leica RXA) and captured by a MicroPublisher 5.0 cooled RTV camera (QImaging).

### Measurement of GAs

Harvested tissues were first ground in liquid nitrogen and used to analyze GAs. GAs were extracted in cold methanol:isopropanol:acetic acid (20:79:1, v/v/v) from 100 mg samples spiked with deuterium-labeled internal standards of GA_1_ (D2-GA1, Olkemim Ltd.). After centrifugation at 16,000 g, the supernatants were collected and extraction of pellet was repeated. The pooled supernatants were evaporated and the resulting pellet was redissolved in 200 μl of 30% methanol. Chromatographic separation of metabolites was accomplished using a 3C18-EP-120 column (0.5 mm × 100 mm, Eksigent) with a mobile gradient of 85% solvent A (0.1% acetic acid in HPLC-grade water, v v^−1^) to 95% solvent B (0.1% acetic acid in 90% acetonitrile, v v^−1^) in 6 min at a flow rate of 15 μl min^−1^. A 6500-QTRAP (AB Sciex) was used to acquire MS spectra. Parameters for analysis were set as follows: ESI in the negative mode (TurboIonSpray), capillary voltage −4,500, nebulizer gas 25 arbitrary units (a.u.), heater gas 25 a.u., curtain gas 10 a.u., collision activation dissociation −2, temperature 250 °C. Gibberellins GA_1_, GA_3_ and GA_4_ were detected using multiple reaction monitoring (MRM) transitions that were optimized using the standards (GA_1_ and GA_4_, Olkemim Ltd; GA_3_, Sigma) and the deuterium-labeled standard. Concentrations were determined from standard curves of known GA concentrations.

### RNA isolation

Harvested tissues were ground in liquid nitrogen, and Qiagen RNeasy kit (Qiagen) was used to isolate the total RNA. DNase I treatment was applied to remove any DNA contamination.

### 454 sequencing

Isolated total RNA was sent to the North Carolina State University Genomic Sciences Laboratory for library preparation and sequencing. RNA quality and concentration were first checked on the Agilent Bioanalyzer 2100 (Agilent Technologies). About 2 μg of total RNA was used for cDNA library preparation using a combination of three kits-Mint-2 cDNA Synthesis Kit (SK005, Evrogen), Trimmer Direct cDNA Normalization Kit (NK002, Evrogen) and GS FLX Titanium Rapid Library Preparation Kit (05608228001, Roche) according to the manufacturer’s protocol. The library was run on the Roche GS FLX (Roche Applied Science) and sequenced. The data was generated using GS De Novo Assembler software (Roche Applied Science). The data were summarized in Supplementary Table S1a.

### Illumina sequencing

The same total RNA extraction method for 454 sequencing was also used to prepare for the RNA samples for Illumina Sequencing performed at North Carolina State University Genomic Sciences Laboratory. The MG and MW RNA-Seq library constructions were carried out using the Illumina TruSeq RNA sample preparation kit while the VG and HG RNA-Seq libraries were constructed using NEBNext Poly(A) mRNA Magnetic Isolation Module (E7490S, New England Biolabs Inc.), NEBNext Ultra Directional RNA Library Prep Kit for Illumina (E7420L, New England Biolabs Inc.), and indexed with the NEBNext Mulitplex Oligos for Illumina (E7335S, New England Biolabs Inc.). All procedures for library construction followed the protocol provided by the vendor. The quantity and qualities of the resulted libraries were verified with a high sensitive DNA kit on the Agilent Bioanalyzer 2100 (Agilent Technologies) on an Agilent RNA 6000 Nano Chip. Libraries were constructed with specific library indexes and pooled in equal molar ratio. The sequencing reactions were run on the Illumina GAIIx with single-end 72 bp for the MG and MW samples and HiSeq 2000 with single-end 100 bp for the VG and HG samples. The Consensus Assessment of Sequence and Variation (CASAVA) software (Illumina) was used to remove adaptor sequences, nucleotide library indexes and generate fastq files. The leaf sectors of MG and MW Illumina data used to build de novo transcriptome assembly were summarized in Supplementary Table S1b, which includes individual MG and MW RNA samples as well as an equal mixture of both. The sequencing results of three VG and three HG libraries were summarized in Supplementary Table S5a.

### Transcript assembly with both 454 and Illumina reads

All sequencing data from 454 and three ‘Marble Queen’ leaf Illumina reads were used to create an assembly using CLC Genomics Workbench 5.0 with the default parameters except for a minimum contig length of 50 bp. The reads were further remapped to the assembled contigs for refinement by CLC Genomics Workbench 5.0. The summary of assembly data is listed in Supplementary Table S1c.

### Functional annotation

The longest ORF was used as a coding sequence for each assembled unique sequence. For functional annotation, the unique sequences were subjected to BLASTX to the NR and TAIR v10 protein database^48^ with a minimum value of 1e-5 and the best hit was assigned. Interproscan 5^49^ was utilized for GO annotation. Other annotations were conducted based on online resources, including Gene Family Annotation (http://www.arabidopsis.org/ browse/genefamily/), Transcription Factor Family (http://datf.cbi.pku.edu.cn/), ROS Enzyme and Reaction (http://biology.unt.edu/ros/pages/genelist.htm), Hormone Function (http://ahd.cbi.pku.edu.cn/) and KEGG annotation (http://www.genome. jp/tools/kaas/).

### Ortholog identification and phylogenetic tree construction

The phylogenetic tree was constructed following the Hal analysis^50^. Briefly, the OrthoMCL v1.4^51^ was applied to identify candidate orthologs from 20 selected plant genomes and *E. aureum* gene set. The All-vs-all blastp implemented in OrthoMCL was executed with a cutoff e-value of 1e-5 by the MCL clustering across a range of inflation parameters (1.1, 1.2, 1.3, 1.4, 1.5, 1.7, 2.0, 2.5, 3.0, 3.5, 4.0, 4.5 5.0) to accommodate diverse evolving genes. After MCL clustering, redundant clusters were removed. Only the orthologous clusters which contained one or no ortholog per plant species and over 50% of taxa contained at least one ortholog were retained for phylogenetic tree construction. For each single copy cluster, multiple sequence alignments were generated using MAFFT^52^. To optimize the alignment for further tree construction, Gblocks^53^ was utilized to remove poorly aligned positions and highly divergent regions. The minimum length of a block was set to 5, and the maximum allowed number of contiguous non-conserved positions was 8. All trimmed alignments were concatenated into a super-alignment with a Perl script. The maximum likelihood phylogenomic tree was built using RAxML^54^ with the PROTGAMMAWAG model of evolution^55^. A bootstrapping with 1,000 replicates was employed and *A. trichopoda* was taken as an out-group tree construction. The tree was drawn by FigTree (http://tree.bio.ed.ac.uk/software/figtree/).

### DEG analysis of VG and HG plants

For DEG analysis of VG and HG plants, RNA-Seq data were obtained using Illumina sequencing platform described above. The sequencing raw reads (Supplementary Table S5a) were mapped back to the assembled contig sequences by Bowtie^56^. About 26% of the sequencing reads that failed to map to a corresponding contig were further assembled using Trinity^22^ and the assembled results were listed in Supplementary Table S5b. The differential expression analysis was conducted by edgeR^57^ with the false discovery rate (FDR) <0.05. Reciprocal blast^58^ using BLASTX and TBLASTN approaches was conducted to selectively search the ortholog gene contigs matching to 147 Arabidopsis flower-related genes and 24 GA biosynthesis genes. Their differential expression analysis was summarized in Supplementary Tables S4 and S6. All matched contigs with gene name information and BLAST hit scores were listed in Supplementary Table S7.

### QRT-PCR and RT-PCR

First strand cDNA was made using the High-Capacity cDNA Reverse Transcription kit (4368814, Applied Biosystems) according to manufacturer’s instructions. For RT-PCR, the reaction was carried out with *Taq* DNA polymerase (D1806, Sigma) using a thermocycler (Biometra). Each 25 μl PCR reaction contained cDNA made from the original 20 ng of RNA together with 1x PCR reaction buffer, 300 nM of each primer, 2 mM MgCl_2_, 0.2 mM dNTP and 1.25U of Taq DNA polymerase. For qRT-PCR, the Power SYBR Green PCR Master mix (4367659, Applied Biosystems) was used. The reactions and fluorescent signal detections were performed under the 7500-Fast Real-Time PCR system (Applied Biosystems). Each sample was assayed in triplicates. The calculation of Ct value was based on Pfaffl^59^. The dCt was a relative expression level compared to the internal control gene *18S rRNA*. The primer for *18S rRNA* was from the Ambion^®^ QuantumRNA™ 18S Internal Standard kit (AM1716, Invitrogen). The fold change of transcript abundance between two samples was calculated by comparing their dCt values (ddCt) in which one ddCt value represents two-fold change. Data from three sets of biological samples were averaged. The information of primer sequences for specific genes is listed in Supplementary Table S8.

### Cloning genomic DNA sequence of *EaLFY*

Since *EaLFY*, an ortholog of Arabidopsis *LFY* (AT5G61850), was not found in initial transcriptomic data nor in analyzed sequence data derived from VG and HG plants by homology search, PCR amplification was used to amplify partial *EaLFY* in *E. aureum*. Based on conserved region of *LFY* exon 3 from 19 monocot and dicot species, two degenerated primers EaLFYF/EaLFYR (Supplementary Table S8) were designed to amplify 294 bp of *EaLFY*. Genomic DNA was isolated from young leaves by DNeasy Plant Mini Kit (69104, Qiagen). Each 25 μl PCR reaction contained 100 ng genomic DNA, together with 1x PCR reaction buffer, 300 nM of each primer, 2 mM MgCl_2_, 0.2 mM dNTP and 1.25U of Taq DNA polymerase. PCR products were cloned into a pCR2.1 vector for sequencing. Amplified 294 bp *EaLFY* gene fragment (Accession #: KP984525, Supplementary Fig. S4b) could be translated into 97 amino acids (Supplementary Fig. S4c). Its amino acid sequences shared 86% identity with those of Arabidopsis *LFY* in the conserved region (Supplementary Fig. S4d). Cloned genomic DNA sequence was used to design primers EaLFYcDNAF3/EaLFYcDNAR3 (Supplementary Table S8) for measuring its expressions under different conditions by RT-PCR.

### Statistical analyses

For analyses of leaf sizes and GA contents, all data were presented as means ± standard deviation (s.d.). Comparisons between VG and HG plants were performed using Student’s *t*-test. The asterisk indicates significant differences between two types of plants. Levels of statistical significance were set at *P < 0.05, **P < 0.01.

## Additional Information

**Accession code:** All sequencing raw reads from 454 and Illumina sequencing platforms as well as their assembled contigs were deposited to the NCBI SRA database under the Bioproject accession number PRJNA286034.

**How to cite this article**: Hung, C.-Y. *et al*. Gibberellin deficiency is responsible for shy-flowering nature of *Epipremnum aureum*. *Sci. Rep.* **6**, 28598; doi: 10.1038/srep28598 (2016).

## Supplementary Material

Supplementary Information

Supplementary Tables S2

Supplementary Tables S3

Supplementary Tables S7

Supplementary Tables S8
