# Supplementary material for: Gibberellin deficiency is responsible for shy-flowering nature of Epipremnum aureum
Source: Sci Rep. 2016 Jun 27;6:28598. doi: 10.1038/srep28598 (PMC4921968; doi:10.1038/srep28598)
Supplement: Supplementary Information [file srep28598-s1.pdf]

Supplementary Information

Gibberellin deficiency is responsible for shy-flowering nature of *Epipremnum aureum*

Chiu-Yueh Hung[1†], Jie Qiu[2†], Ying-Hsuan Sun[3], Jianjun Chen[4], Farooqahmed S. Kittur[1], Richard J. Henny[4], Gulei Jin[2], Longjiang Fan[2*], Jiahua Xie[1*]

[*]: Correspondence and requests for materials should be addressed to J.X. (jxie@nccu.edu) or L.F. (fanlj@zju.edu.cn).

This PDF file includes

Supplementary Figs. 1 to 5

Supplementary Tables S1, S4-S6

Other Supplementary Material for this manuscript includes the following:

Supplementary Table S2. The 20 genomes of angiosperm species used in this study

Supplementary Table S3. Single copy orthologs from *E. aureum* and other 20 angiosperm species

Supplementary Table S7. Full names of Arabidopsis genes used and their corresponding *E. aureum* orthologs

Supplementary Table S8. Primers for qRT-PCR, PCR and RT-PCR

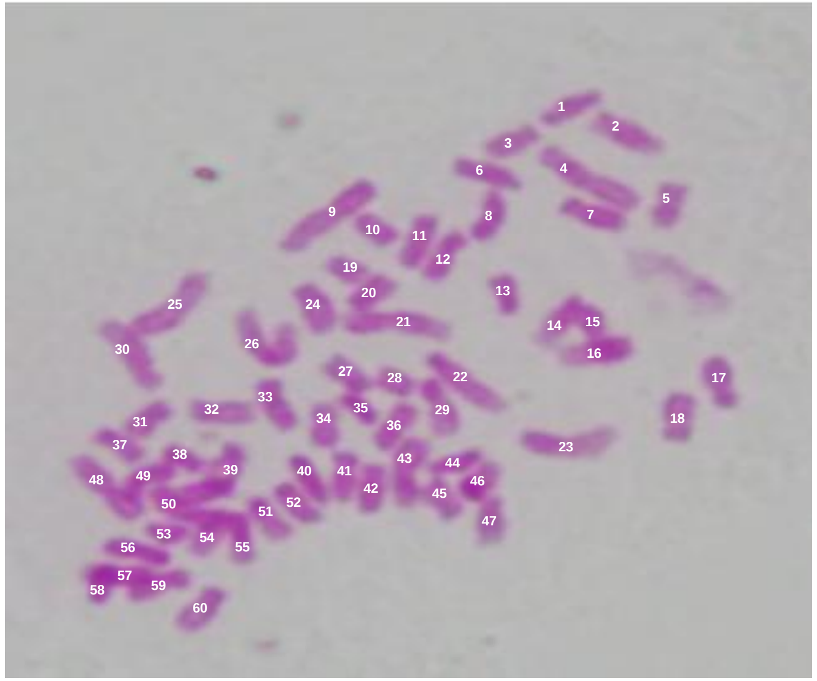

Supplementary Figure S1. Chromosome number. A total of 60 metaphase chromosomes were observed in a root-tip cell (x 2000).

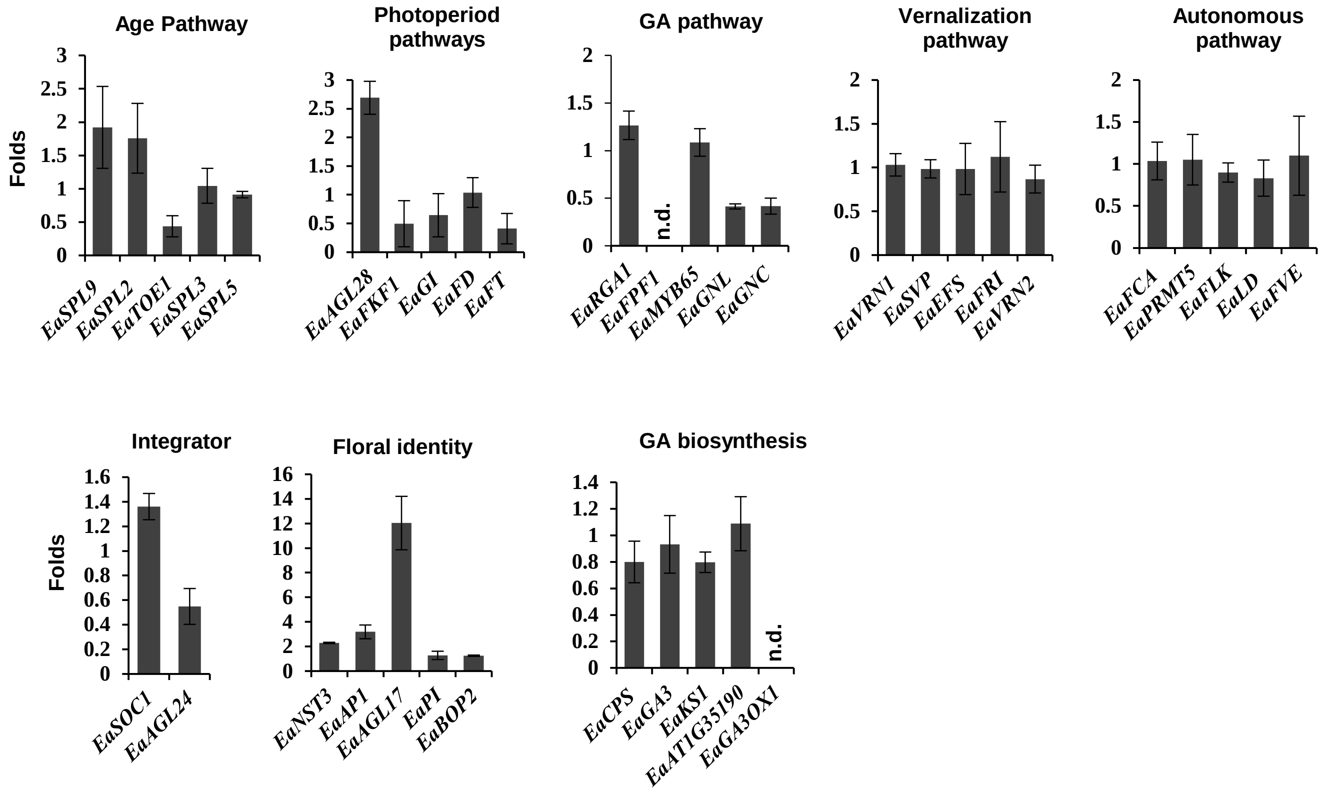

Supplementary Figure S2. Results of qRT-PCR on selected genes from each floral inductive pathway or group for validating the DEG results in VG compared to HG plants. Data plotted are the fold changes representing the relative difference in expression levels between VG plants and HG plants (as 1). Data shown is the average (n=3) ± s.d.. n.d.: not detected.

a **b**

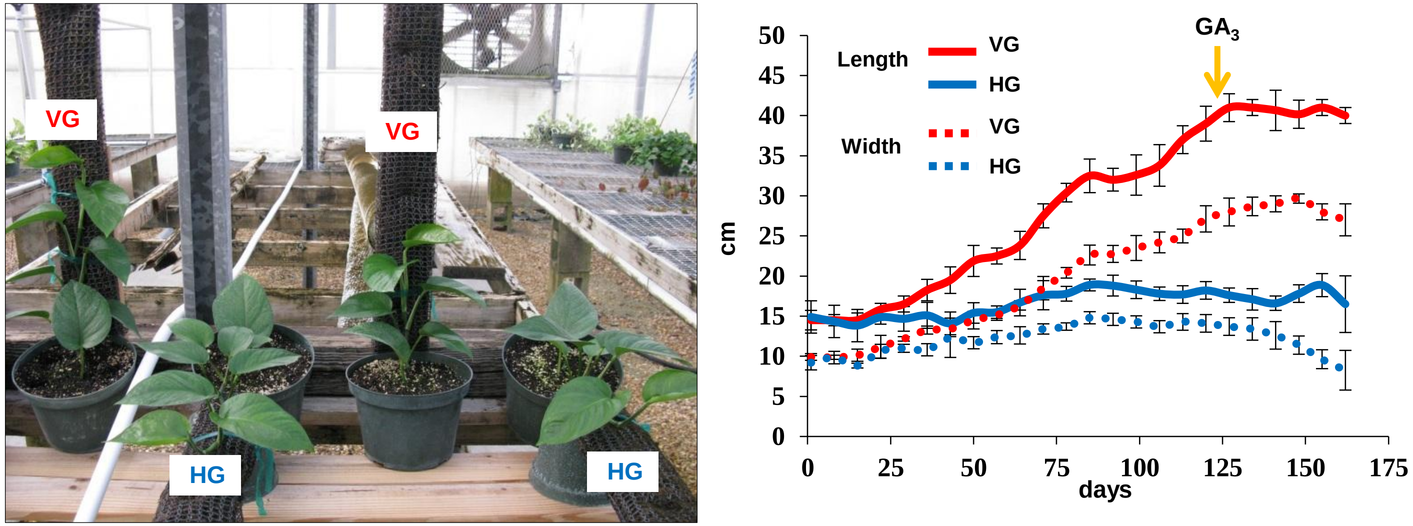

Supplementary Figure S3. VG and HG plants used for GA_3 treatment. (**a**) Green 'Jade' plants with 5 to 6 leaves propagated by cutting were used to set up on totem poles for vertical and horizontal growth studies. They were grown in a shaded greenhouse at the University of Florida Mid-Florida Research and Education Center, Apopka, FL, USA. (**b**) Leaf length (cm) and width (cm) of the latest fully expanded leaf were recorded weekly. Data plotted were the average (n=5) ± s.d.. Arrow indicates the time of GA_3 treatment.

a >LEAFY (Arabidopsis Accession #: NP_200993.1)

MDPEGFTSGLFRWNPTRALVQAPPPVPPPLQQQPVTPQTA
AFGMRLGGLEGLFGPYGIRFYTAAKIAELGFTASTLVGMK
DEELEEMMNSLSHIFRWELLVGERYGIKAAVRAERRRLQ
EEEEEESSRRRHLLLSAAGDSGTHHALDALSQEGLSEEPV
QQQDQTDAAGNNGGGGSGYWDAGQGKMKKQQQQRRR
KKPMLTSVETDEDVNEGEDDDGMDNGNGGSGLGTERQR
EHPFIVTEPGEVARGKKNGLDYLFHLYEQCREFLLQVQTI
AKDRGEKCPTKVTNQVFRYAKKSGASYINKPKMRHYVH
CYALHCLDEEASNALRRAFKERGENVGSWRQACYKPLV
NIACRHGWDIDAVFNAHPRLSIWYVPTKLRQLCHLERNN
AVAAAAALVGGISCTGSSTSGRGGCGGDDLRF

b >*EaLFY* (*E. aureum* Accession #: KP984525)

GACGAATCAGGTGTTCAGGTACGCGAAG
GAGGCAGGAGCGAGCTACATCAACAAG
CCCAAGATGCGGCACTACGTCCACTGCT
ACGCCCTGCACTGCCTGGACGAGGAGGC
GTCCAACGCACTGCGGCGGGCGTTCAAG
GAGAAGGGCGAGAACGTGGGCGCGTGG
CGGCAGGCCTGCTACCAGCCCCTGGTGG
CCATGTCCGCCCGCCACGCCTGGGACGT
CGACGCCCTCTTCAACTCCCACCCCCGTC
TCGCCATCTGGTACGTCCCCACCAAACTG
CGCCAGCTGTGCCA

c >EaLFY (97 aa)

TNQVFRYAKEAGASYIN
KPKMRHYVHCYALHCL
DEEASNALRRAFKEKGE
NVGAWRQACYQPLVA
MSARHAWDVDALFNSH
PRLAIWYVPTKLRQLC

d

```
AtLFY TNQVFRYAKKSGASYINKPKMRHYVHCYALHCLDEEASNALRRAFKERGENVGSWRQACY 60
EaLFY TNQVFRYAKEAGASYINKPKMRHYVHCYALHCLDEEASNALRRAFKEKGENVGAWRQACY 60
      *********::************************************:*****:******
AtLFY KPLVNIACRHGWDIDAVFNAHPRLSIWYVPTKLRQLC 97
EaLFY QPLVAMSARHAWDVDALFNSHPRLAIWYVPTKLRQLC 97
      :*** ::.**.**:**:**:****:************
```

Supplementary Figure S4. Amplified partial genomic DNA sequence of *EaLFY*. (**a**) The underlined region of Arabidopsis LEAFY amino acid sequence represents the conserved region of *LFY* exon 3 among 19 monocot and dicot species used for designing two degenerated primers EaLFYF/EaLFYR. (**b**) Amplified 294 bp *EaLFY* genomic sequences. The first and last codons are underlined. (**c**) Derived amino acid sequence of EaLFY. (**d**) CLUSTAL 2.1 multiple sequence alignment of Arabidopsis LEAFY (AtLFY) and EaLFY showing 86% identity.

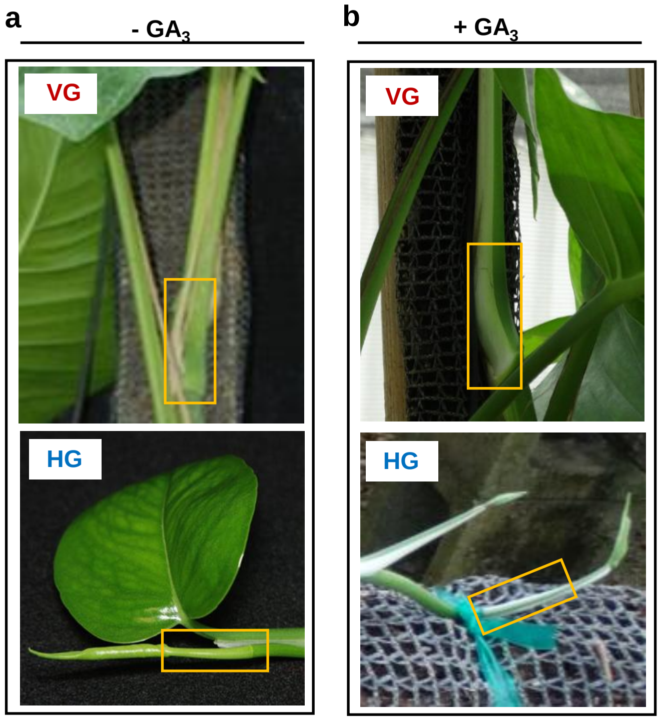

Supplementary Figure S5. Photographs showing regions used for harvesting young shoots. (**a**) Young shoots of VG and HG plants without GA_3 treatment (-GA_3) were used for both gene expression analysis and GA measurement. (**b**) Young shoots of VG and HG plants with GA_3 treatment (+ GA_3) were used for gene expression analysis.

Supplementary Table S1a. Summary of transcriptomic reads by 454 sequencing platform[a]

Number of reads	579,096
Minimum read length (bp)	40
Maximum read length (bp)	1,252
Mean read length before trimming (bp)	388
Total length (bp)	224,619,644

[a]**:** Mixed RNAs prepared from young shoots including leaves, petioles and stems as well as young roots were used.

Supplementary Table S1b. Summary of Illumina clean reads of 'Marble Queen' leaves[a]

	MG	MW	Mixed RNA sample	Total
Reads number	35,067,199	32,265,993	28,478,946	95,812,138
Bases in total	2,384,569,532	2,194,087,524	1,452,426,246	6,031,083,302

[a]: MG: 'Marble Queen' green section tissues; MW: 'Marble Queen' white section tissues. Mixed RNA sample: equal amount of total RNA from MG and MW was used.

Supplementary Table S1c. Summary of transcripts assembled

Count	454+Illumina (leaf)
Total reads	96,391,234
Average read length (bp)	64.9
Total bases	6,255,702,946
Reads aligned	71,839,267
Average aligned read length (bp)	62.0
Aligned bases	4,732,256,144
Number of contigs	41,059
Average contig length (bp)	1,049
N50 contig size (bp)	1,135
Largest contig size (bp)	13,055

Supplementary Table S4. DEG analysis results of 147 orthologs of *Arabidopsis* flower-related genes in VG and HG plants[a].

At gene name	*At* Locus ID	*Ea* gene name	VG	HG	LogFC	LogCPM	*P* value	FDR
Age pathway								
TOE1 **[b,c]**	**AT2G28550.1**	***EaTOE1*** [d]	**429.0**	**785.3**	**-1.09**	**4.60**	**7.17E-09**	**8.68E-07**
SPL9	**AT2G42200.1**	***EaSPL9*** [d]	**358.3**	**166.0**	**0.94**	**3.29**	**1.90E-06**	**0.000136**
SPL15	**AT3G57920.1**	***EaSPL15***	**358.3**	**166.0**	**0.94**	**3.29**	**1.90E-06**	**0.000136**
SPL2	**AT5G43270.1**	***EaSPL2*** [d]	**885.3**	**450.0**	**0.78**	**4.64**	**3.45E-05**	**0.001741**
SPL10	**AT1G27370.1**	***EaSPL10***	**885.3**	**450.0**	**0.78**	**4.64**	**3.45E-05**	**0.001741**
SPL11	**AT1G27360.1**	***EaSPL11***	**885.3**	**450.0**	**0.78**	**4.64**	**3.45E-05**	**0.001741**
SPL8	**AT1G02065.1**	***EaSPL8*** [e]	**57.3**	**24.0**	**1.03**	**0.67**	**0.000193**	**0.007546**
SPL8	AT1G02065.1	*EaSPL8* [e]	30.0	14.3	0.86	-0.14	0.009192	0.144582
SPL6	AT1G69170.1	*EaSPL6* [e]	111.0	73.3	0.36	1.82	0.116357	0.605206
SPL5	AT3G15270.1	*EaSPL5* [d]	607.7	470.7	0.18	4.37	0.344013	0.894944
SPL4	AT1G53160.1	*EaSPL4*	607.7	470.7	0.18	4.37	0.344013	0.894944
AP2	AT4G36920.1	*EaAP2*	218.0	207.3	-0.13	3.04	0.506005	0.981455
SMZ/SNZ	AT2G39250.1	*EaSMZ/SNZ*	218.0	207.3	-0.13	3.04	0.506005	0.981455
SPL13	AT5G50670.1	*EaSPL13* [e]	309.7	252.7	0.08	3.43	0.681925	1
SPL13	AT5G50670.1	*EaSPL13* [e]	72.3	67.3	-0.10	1.45	0.693374	1
SPL3	AT2G33810.1	*EaSPL3* [d]	2876.0	2372.7	0.08	6.65	0.674808	1
SPL6	AT1G69170.1	*EaSPL6* [e]	309.7	249.3	0.11	3.41	0.59873	1
SPL8	AT1G02065.1	*EaSPL8* [e]	111.7	91.0	0.07	2.00	0.789434	1
TOE2	AT5G60120.1	*EaTOE2*	886.0	801.7	-0.08	5.02	0.670706	1
Autonomous pathway								
FPA	AT2G43410.1	*EaFPA*	130.3	94.7	0.26	2.13	0.239574	0.800147
REF6	AT3G48430.1	*EaREF6*	378.0	384.7	-0.22	3.88	0.258182	0.819836
FCA	AT4G16280.1	*EaFCA* [d]	484.3	402.0	0.07	4.08	0.706796	1
PRMT5	AT4G31120.1	*EaPRMT5* [d]	3161.7	2655.0	0.07	6.79	0.707172	1
FLD	AT3G10390.1	*EaFLD*	805.7	731.0	-0.06	4.88	0.748473	1
FLK	AT3G04610.1	*EaFLK* [d]	3104.3	2674.3	0.04	6.78	0.846248	1
FPA	AT2G43410.1	*EaFPA*	914.3	819.0	-0.04	5.05	0.826375	1
FVE	AT2G19520.1	*EaFVE* [d]	4268.7	3646.7	0.03	7.23	0.879229	1
FY	AT5G13480.1	*EaFY*	952.0	775.3	0.09	5.04	0.617909	1
LD	AT4G02560.1	*EaLD* [d]	890.7	776.3	0.00	5.00	0.982486	1
Floral identity genes								
AGL17	**AT2G22630.1**	***EaAGL17*** [d]	**112.7**	**8.3**	**3.51**	**1.17**	**6.07E-31**	**1.53E-27**
AP1	**AT1G69120.1**	***EaAP1*** [d]	**129.7**	**38.3**	**1.55**	**1.67**	**4.90E-11**	**8.67E-09**
AGL2	**AT5G15800.1**	***EaAGL2***	**129.7**	**38.3**	**1.55**	**1.67**	**4.90E-11**	**8.67E-09**
AGL3	**AT2G03710.1**	***EaAGL3***	**129.7**	**38.3**	**1.55**	**1.67**	**4.90E-11**	**8.67E-09**
AGL4	**AT3G02310.1**	***EaAGL4***	**129.7**	**38.3**	**1.55**	**1.67**	**4.90E-11**	**8.67E-09**
AGL6	**AT2G45650.1**	***EaAGL6***	**129.7**	**38.3**	**1.55**	**1.67**	**4.90E-11**	**8.67E-09**
AGL9	**AT1G24260.1**	***EaAGL9***	**129.7**	**38.3**	**1.55**	**1.67**	**4.90E-11**	**8.67E-09**
AGL10/CAL1	**AT1G26310.1**	***EaAGL10/EaCAL1***	**129.7**	**38.3**	**1.55**	**1.67**	**4.90E-11**	**8.67E-09**

PI	**AT5G20240.1**	***EaPI*** [d]	**62.7**	**30.0**	**0.88**	**0.85**	**0.000569**	**0.018433**
FUL/AGL8	**AT5G60910.1**	***EaFUL/EaAGL8***	**16.7**	**5.0**	**1.51**	**-1.04**	**0.000937**	**0.027242**
NST1	**AT2G46770.1**	***EaNST1***	**36.0**	**14.3**	**1.07**	**0.04**	**0.00117**	**0.03251**
NST3	**AT1G32770.1**	***EaNST3*** [d]	**36.0**	**14.3**	**1.07**	**0.04**	**0.00117**	**0.03251**
DDF1	AT1G12610.1	*EaDDF1*	76.7	101.7	-0.63	1.83	0.004167	0.083805
AG	AT4G18960.1	*EaAG*	557.7	339.3	0.53	4.08	0.005592	0.103145
AGL11/STK	AT4G09960.1	*EaAGL11/EaSTK*	557.7	339.3	0.53	4.08	0.005592	0.103145
AGL14	AT4G11880.1	*EaAGL14*	557.7	339.3	0.53	4.08	0.005592	0.103145
AGL15	AT5G13790.1	*EaAGL15*	557.7	339.3	0.53	4.08	0.005592	0.103145
AGL19	AT4G22950.1	*EaAGL19*	557.7	339.3	0.53	4.08	0.005592	0.103145
AGL42	AT5G62165.1	*EaAGL42*	557.7	339.3	0.53	4.08	0.005592	0.103145
AGL71	AT5G51870.1	*EaAGL71*	557.7	339.3	0.53	4.08	0.005592	0.103145
AGL72	AT5G51860.1	*EaAGL72*	557.7	339.3	0.53	4.08	0.005592	0.103145
AGL18	AT3G57390.1	*EaAGL18*	557.7	339.3	0.53	4.08	0.005592	0.103145
AP3	AT3G54340.1	*EaAP3*	4.7	9.3	-1.18	-1.53	0.022435	0.256333
LLP12/CRN/SOL2	AT5G13290.1	*EaLLP12/EaCRN/EaSOL2*	147.7	162.0	-0.34	2.60	0.093812	0.551095
LMI1	AT5G03790.1	*EaLMI1* [e]	399.7	415.7	-0.24	3.98	0.221318	0.781607
LMI1	AT5G03790.1	*EaLMI1* [e]	799.0	794.7	-0.19	4.94	0.297594	0.855573
BOP2	AT2G41370.1	*EaBOP2* [d]	158.0	120.0	0.20	2.43	0.351421	0.898767
AGL12	AT1G71692.1	*EaAGL12*	181.0	177.0	-0.16	2.79	0.442208	0.952396
BOP1	AT3G57130.1	*EaBOP1*	250.3	201.3	0.12	3.12	0.563205	0.998487
CLI1/ RPK2/TOAD2	AT3G02130.1	*EaCLI1/ EaRPK2/EaTOAD2* [e]	233.3	213.3	-0.08	3.12	0.685353	1
CLI1/ RPK2/TOAD2	AT3G02130.1	*EaCLI1/ EaRPK2/EaTOAD2* [e]	224.3	200.3	-0.02	3.03	0.954001	1
FLY1	AT4G28370.1	*EaFLY1*	171.3	137.7	0.08	2.58	0.734286	1
FLY2	AT2G20650.1	*EaFLY2*	171.3	137.7	0.08	2.58	0.734286	1
IFL	AT5G60690.1	*EaIFL*	1360.3	1143.0	0.04	5.58	0.850185	1
LMI1	AT5G03790.1	*EaLMI1* [e]	5118.0	4442.0	-0.01	7.52	0.975997	1
LMI1	AT5G03790.1	*EaLMI1* [e]	264.3	228.7	-0.00	3.26	0.992052	1
LMI1	AT5G03790.1	*EaLMI1* [e]	359.3	319.3	-0.04	3.71	0.840753	1
PI	AT5G20240.1	*EaPI*	296.3	247.3	0.06	3.38	0.746256	1
SEU	AT1G43850.1	*EaSEU*	1034.7	879.0	0.03	5.19	0.877699	1
TFL1	AT5G03840.1	*EaTFL1*	152.3	126.7	0.10	2.40	0.61737	1
WDR55	AT2G34260.1	*EaWDR55*	1109.3	922.0	0.07	5.26	0.716504	1
LFY	AT5G61850	*EaLFY*	Not found					
Integrator genes								
SOC1	AT2G45660.1	*EaSOC1* [d]	557.7	339.3	0.53	4.08	0.01	0.103145
AGL24	AT4G24540.1	*EaAGL24* [d]	667.7	660.3	-0.18	4.67	0.36	0.901101
Photoperiod pathway								
MFT	**AT1G18100.1**	***EaMFT***	**28.0**	**1.0**	**4.43**	**-0.72**	**3.91E-15**	**1.46E-12**
FKF1	**AT1G68050.1**	***EaFKF1*** [d,e]	**628.0**	**1201.3**	**-1.09**	**5.21**	**5.52E-09**	**6.85E-07**
AGL28	**AT1G01530.1**	***EaAGL28*** [d]	**1831.7**	**844.7**	**0.88**	**5.65**	**2.01E-06**	**0.000143**
FT	**AT1G65480.1**	***EaFT****	**16.7**	**39.0**	**-1.39**	**0.21**	**5.95E-06**	**0.000374**

TSF	**AT4G20370.1**	***EaTSF***	**16.7**	**39.0**	**-1.39**	**0.21**	**5.95E-06**	**0.000374**
FKF1	**AT1G68050.1**	***EaFKF1*** [e]	**272.3**	**388.7**	**-0.67**	**3.72**	**0.000586**	**0.018859**
GI	**AT1G22770.1**	***EaGI*** [d,e]	**4190.3**	**5680.0**	**-0.60**	**7.61**	**0.000997**	**0.028733**
GI	**AT1G22770.1**	***EaGI*** [e]	**6124.7**	**8277.7**	**-0.59**	**8.15**	**0.001273**	**0.034711**
FD	AT4G35900.1	*EaFD* [d,e]	65.0	33.3	0.76	0.94	0.003268	0.070659
CO	AT5G15840.1	*EaCO*	11.7	16.7	-0.73	-0.69	0.045724	0.381351
COP1	AT2G32950.1	*EaCOP1*	361.0	389.3	-0.34	3.88	0.073417	0.488922
CDF1	AT5G62430.1	*EaCDF1*	278.3	180.0	0.35	3.11	0.082985	0.520126
PFT1	AT1G25540.1	*EaPFT1*	2007.0	1434.0	0.27	6.02	0.138052	0.646008
PhyA	AT1G09570.1	*EaPhyA*	2566.3	2633.7	-0.25	6.65	0.173625	0.709623
PhyB	AT2G18790.1	*EaPhyB* [e]	374.0	385.7	-0.23	3.89	0.229679	0.789765
FTIP1	AT5G06850.1	*EaFTIP1*	357.7	265.7	0.22	3.58	0.270946	0.835217
CRY1	AT4G08920.1	*EaCRY1*	3929.3	3691.0	-0.11	7.20	0.538607	0.991724
ATC	AT2G27550.1	*EaATC*	152.3	126.7	0.10	2.40	0.61737	1
BFT	AT5G62040.1	*EaBFT*	152.3	126.7	0.10	2.40	0.61737	1
CRY2	AT1G04400.1	*EaCRY2*	2729.3	2274.0	0.07	6.58	0.700354	1
ELF6	AT5G04240.1	*EaELF6*	196.7	173.7	-0.04	2.85	0.835227	1
FD	AT4G35900.1	*EaFD* [e]	109.7	92.0	0.04	1.98	0.893786	1
ADO1/FKL2	AT5G57360.1	*EaADO1/EaFKL2* [e]	660.0	544.3	0.08	4.52	0.68718	1
ADO1/FKL2	AT5G57360.1	*EaADO1/EaFKL2* [e]	788.3	699.0	-0.02	4.83	0.933448	1
PhyB	AT2G18790.1	*EaPhyB* [e]	362.7	312.7	0.02	3.70	0.908673	1
PhyC	AT5G35840.1	*EaPhyC*	1761.3	1535.7	0.02	5.98	0.900044	1
SPA1	AT2G46340.1	*EaSPA1*	283.0	237.3	0.06	3.32	0.773969	1
TEM1	AT1G25560.1	*EaTEM1*	97.0	85.3	0.03	1.83	0.845726	1
TEM2/RAV2	AT1G68840.1	*EaTEM2/EaRAV2*	97.0	85.3	0.03	1.83	0.845726	1
Vernalization pathway								
BRM	AT2G46020.1	*EaBRM* [e]	397.0	442.0	-0.37	4.04	0.054448	0.421789
BRM	AT2G46020.1	*EaBRM* [e]	556.0	604.7	-0.32	4.49	0.08628	0.528592
EMF1	AT5G11530.1	*EaEMF1*	24.0	28.7	-0.48	0.14	0.104773	0.580091
ELF3	AT2G25930.1	*EaELF3*	222.0	238.3	-0.28	3.17	0.160058	0.68568
VRN1	AT3G18990.1	*EaVRN1* [d,e]	61.7	66.0	-0.29	1.34	0.230591	0.789765
FLM/ AGL27	AT1G77080.2	*EaFLM/ EaAGL27*	667.7	660.3	-0.17	4.67	0.356247	0.901101
SVP	AT2G22540.1	*EaSVP* [e]	667.7	660.3	-0.17	4.67	0.356247	0.901101
FLC /AGL25	AT5G10140.1	*EaFLC /EaAGL25*	667.7	660.3	-0.17	4.67	0.356247	0.901101
MAF2	AT5G65050.1	*EaMAF2*	667.7	660.3	-0.17	4.67	0.356247	0.901101
MAF3	AT5G65060.1	*EaMAF3*	667.7	660.3	-0.17	4.67	0.356247	0.901101
MAF4/AGL69	AT5G65070.1	*EaMAF4/EaAGL69*	667.7	660.3	-0.17	4.67	0.356247	0.901101
MAF5/AGL68	AT5G65080.1	*EaMAF5/EaAGL68*	667.7	660.3	-0.17	4.67	0.356247	0.901101
ELF7	AT1G79730.1	*EaELF7*	299.0	291.7	-0.17	3.51	0.373069	0.913347
SWI	AT2G33610.1	*EaSWI*	322.3	254.3	0.14	3.47	0.480362	0.969466
SYD	AT2G28290.1	*EaSYD*	259.7	246.0	-0.14	3.29	0.487328	0.973101
LDL1/SWP1	AT1G62830.1	*EaLDL1/EaSWP1*	400.0	318.0	0.13	3.78	0.489921	0.974877
BRM	AT2G46020.1	*EaBRM* [e]	1089.3	1040.3	-0.13	5.36	0.494815	0.976935

EFS	AT1G77300.1	*EaEFS* [d]	1494.0	1415.3	-0.12	5.80	0.507713	0.981455
VIP3	AT4G29830.1	*EaVIP3*	333.3	267.7	0.13	3.53	0.518662	0.983689
PIE1	AT3G12810.1	*EaPIE1*	1053.3	839.0	0.11	5.18	0.547718	0.996212
ESD1/ARP6	AT3G33520.1	*EaESD1/EaARP6*	338.7	312.3	-0.11	3.64	0.560551	0.998333
MEA	AT1G02580.1	*EaMEA*	693.7	584.7	0.06	4.63	0.763444	1
ATX2	AT1G05830.1	*EaATX2*	350.0	279.3	0.11	3.60	0.591241	1
UBC1	AT1G14400.1	*EaUBC1*	4390.0	3964.0	-0.04	7.32	0.844097	1
VIP5	AT1G61040.1	*EaVIP5*	1526.3	1264.0	0.08	5.73	0.681785	1
UBC2	AT2G02760.1	*EaUBC2*	4390.0	3964.0	-0.04	7.32	0.844097	1
ELF8	AT2G06210.1	*EaELF8*	2502.3	2222.7	-0.03	6.50	0.872752	1
SVP	AT2G22540.1	*EaSVP* [d,e]	1385.0	1179.7	0.04	5.61	0.832829	1
CLF	AT2G23380.1	*EaCLF*	693.7	584.7	0.06	4.63	0.763444	1
ATX1	AT2G31650.1	*EaATX1*	350.0	279.3	0.11	3.60	0.591241	1
ELF4	AT2G40080.1	*EaELF4*	41.0	34.3	0.05	0.59	0.883057	1
BRM	AT2G46020.1	*EaBRM* [e]	1151.3	1071.3	-0.09	5.42	0.61169	1
BSH/CHE1	AT3G17590.1	*EaBSH/EaCHE1*	1097.7	955.3	0.00	5.28	0.994548	1
VRN1	AT3G18990.1	*EaVRN1* [e]	49.7	45.7	-0.06	0.92	0.837546	1
FIE	AT3G20740.1	*EaFIE*	1116.3	1015.0	-0.05	5.35	0.782469	1
VIL1	AT3G24440.1	*EaVIL1*	459.3	383.3	0.06	4.00	0.746145	1
FRI	AT4G00650.1	*EaFRI* [d,e]	745.0	668.0	-0.05	4.75	0.783395	1
FRI	AT4G00650.1	*EaFRI* [e]	330.3	282.0	0.03	3.55	0.888597	1
SWN/EZA1	AT4G02020.1	*EaSWN/EaEZA1*	693.7	584.7	0.06	4.63	0.763444	1
VRN2	AT4G16845.1	*EaVRN2* [d]	1638.3	1472.7	-0.06	5.89	0.745191	1
EMF1	AT5G11530.1	*EaEMF1*	12.7	11.3	-0.03	-0.90	1	1
SWC6	AT5G37055.1	*EaSWC6*	229.3	211.7	-0.06	3.08	0.787716	1
EMF2	AT5G51230.1	*EaEMF2*	1638.3	1472.7	-0.06	5.89	0.745191	1
VIN3	AT5G57380.1	*EaVIN3*	373.0	334.3	-0.04	3.76	0.855417	1
MSI1	AT5G58230.1	*EaMSI1*	1822.3	1632.7	-0.04	6.04	0.837435	1
VIP4	AT5G61150.1	*EaVIP4*	915.3	804.7	-0.02	5.03	0.931436	1
UBC3	AT5G62540.1	*EaUBC3*	4390.0	3964.0	-0.04	7.32	0.844097	1
ELF5	AT5G62640.1	*EaELF5*	198.7	165.7	0.05	2.81	0.821495	1
GA pathway								
GNL	**AT4G26150.1**	***EaGNL*** [d,e]	**56.0**	**161.0**	**-1.70**	**2.11**	**2.34E-14**	**7.44E-12**
GNC	**AT5G56860.1**	***EaGNC*** [d]	**143.7**	**351.0**	**-1.48**	**3.29**	**2.07E-13**	**5.76E-11**
GNL	**AT4G26150.1**	***EaGNL*** [e]	**79.3**	**163.7**	**-1.25**	**2.28**	**5.55E-09**	**6.87E-07**
JAZ10	AT5G13220.1	*EaJAZ10*	124.0	159.7	-0.59	2.50	0.004614	0.089664
GAI	AT1G14920.1	*EaGAI*	2638.7	1839.3	0.30	6.41	0.1067	0.584401
RGL1	AT1G66350.1	*EaRGL1*	2638.7	1839.3	0.30	6.41	0.1067	0.584401
RGA1	AT2G01570.1	*EaRGA1* [d]	2638.7	1839.3	0.30	6.41	0.1067	0.584401
RGL2	AT3G03450.1	*EaRGL2*	2638.7	1839.3	0.30	6.41	0.1067	0.584401
RGL3	AT5G17490.1	*EaRGL3*	2638.7	1839.3	0.30	6.41	0.1067	0.584401
AtMYB101	AT2G32460.1	*EaMYB101*	109.3	117.7	-0.30	2.14	0.164242	0.693669
AtMYB65	AT3G11440.1	*EaMYB65* [d]	109.3	117.7	-0.30	2.14	0.164242	0.693669

AtMYB33	AT5G06100.1	*EaMYB33*	109.3	117.7	-0.30	2.14	0.164242	0.693669
GID1	AT3G05120.1	*EaGID1*	127.0	132.7	-0.29	2.36	0.172475	0.708781
SPY	AT3G11540.1	*EaSPY*	1342.3	1320.0	-0.16	5.68	0.394737	0.927101
JAZ5	AT1G17380.1	*EaJAZ5*	384.3	304.7	0.13	3.72	0.488642	0.973784
JAZ6	AT1G72450.1	*EaJAZ6*	384.3	304.7	0.13	3.72	0.488642	0.973784
JAZ1	AT1G19180.1	*EaJAZ1*	761.7	658.3	0.01	4.76	0.971806	1
AGL67	AT1G77950.1	*EaAGL67*	49.0	44.7	-0.08	0.90	0.740824	1
PKL	AT2G25170.1	*EaPKL* [e]	799.3	725.3	-0.05	4.87	0.801338	1
PKL	AT2G25170.1	*EaPKL* [e]	2084.3	1813.0	-0.00	6.22	0.989899	1
GRAS family transcription factor	AT2G37650.1	*EaGRAS family transcription factor*	424.3	342.7	0.09	3.88	0.644304	1
FPF1	AT5G24860.1	*EaFPF1* [d]	0.0	0.0	0.00	-3.71	1	1

[a]: *At*: *A. thaliana*; *Ea*: *E. aureum*; FC: fold change; CPM: read counts per million reads; FDR: false discovery rate. [b]: Gene full names and corresponding contigs are listed in supplementary table S7. [c]: FDR<0.05 is bold. [d]: Selected for qRT-PCR analysis. [e]: Genes with multiple matched contigs.

Supplementary Table S5a. Summary of Illumina clean reads of 'Jade' VG and HG shoots[a]

	VG1	VG2	VG3	HG1	HG2	HG3	Total
Reads number	31123913	28548121	26858706	27466625	30513093	21407622	165918080
Unmapped reads	7944793	7344331	7001899	7460861	7886162	5811986	43450032
Bases in total	3066980460	2813639125	2645293654	2703821121	3006510903	2108534014	16344779277
Bases in total (unmapped)	778205536	719570942	685831994	730324520	772839329	569059479	4255831800

[a]: VG: vertical growth; HG: horizontal growth. The Arabic numerals next to VG and HG indicate three different plants used for sequencing.

Supplementary Table S5b. Summary of transcripts assembled from unmapped data

Total reads	43,450,032
Average read length (bp)	68
Total bases	4,255,831,800
Aligned bases	25,002,043
Number of contigs	54,712
Average contig length (bp)	457
N50 contig size (bp)	514
Largest contig size (bp)	8,567

Supplementary Table S6. DEG analysis results of 24 orthologs of *Arabidopsis* GA biosynthesis genes in VG and HG plants[a]

At gene name	*At* Locus ID	*Ea* gene name	VG	HG	LogFC	LogCPM	*P* value	FDR
**AT1G14120.1**[b,c]	**AT1G14120.1**	***EaAT1G14120***	39.7	61.3	**-0.84**	**1.05**	**0.000783**	**0.023595**
AT1G14130.1	AT1G14130.1	*EaAT1G14130*	793.3	897.0	-0.38	5.03	0.043685	0.370909
AT4G21690.1	AT4G21690.1	*EaAT4G21690*	793.3	897.0	-0.38	5.03	0.043685	0.370909
KS1	AT1G79460.1	*EaKS1* [d]	468.7	524.0	-0.34	4.26	0.074051	0.490749
AT3G19010.1	AT3G19010.1	*EaAT3G19010*	261.7	189.3	0.27	3.10	0.17111	0.706718
AT1G35190.1	AT1G35190.1	*EaAT1G35190* [d]	296.3	224.7	0.22	3.32	0.261283	0.823271
GA2ox2	AT1G30040.1	*EaGA2ox2*	45.3	48.3	-0.27	0.90	0.320581	0.875759
GA2ox6	AT1G02400.1	*EaGA2ox6*	45.3	48.3	-0.27	0.90	0.320581	0.875759
AT4G23340.1	AT4G23340.1	*EaAT4G23340*	95.7	73.0	0.20	1.72	0.364453	0.909479
AT5G51310.1	AT5G51310.1	*EaAT5G51310*	7.3	4.3	0.54	-1.76	0.421071	0.940553
GA20ox1	AT4G25420.1	*EaGA20ox1*	19.3	20.0	-0.26	-0.27	0.444194	0.952396
GA20ox2	AT5G51810.1	*EaGA20ox2*	19.3	20.0	-0.26	-0.27	0.444194	0.952396
GA20ox3	AT5G07200.1	*EaGA20ox3*	19.3	20.0	-0.26	-0.27	0.444194	0.952396
GA20ox4	AT1G60980.1	*EaGA20ox4*	19.3	20.0	-0.26	-0.27	0.444194	0.952396
GA20ox5	AT1G44090.1	*EaGA20ox5*	19.3	20.0	-0.26	-0.27	0.444194	0.952396
KAO1	AT1G05160.1	*EaKAO1*	58.0	57.7	-0.18	1.19	0.480754	0.969466
KAO2	AT2G32440.1	*EaKAO2*	58.0	57.7	-0.18	1.19	0.480754	0.969466
AT1G80330.1	AT1G80330.1	*EaAT1G80330*	0.0	0.0	0.00	-3.71	1	1
CCR1	AT1G15950.1	*EaCCR1*	338.3	294.7	-0.03	3.61	0.86192	1
CCR2	AT1G80820.1	*EaCCR2*	338.3	294.7	-0.03	3.61	0.86192	1
CPS	AT4G02780.1	*EaCPS* [d]	124.7	119.0	-0.11	2.24	0.609502	1
GA3	AT5G25900.1	*EaGA3* [d]	468.0	399.0	0.06	4.06	0.756214	1
GA3ox1	AT1G15550.1	*EaGA3ox1*[d]	0.0	0.0	0.00	-3.71	1	1
GA3ox2	AT1G80340.1	*EaGA3ox2*	0.0	0.0	0.00	-3.71	1	1

[a]: *At*: *A. thaliana*; *Ea*: *E. aureum*; FC: fold change; CPM: read counts per million reads; FDR: false discovery rate. [b]: Gene full names and corresponding contigs are listed in supplementary table S7. [c]: FDR<0.05 is bold. [d]: Selected for qRT-PCR analysis.
